# Cross-cultural adaptation and psychometric validation of the ISBAR structured handover observation tool for ICU-to-ward patient transfer in China

**DOI:** 10.1371/journal.pone.0347947

**Published:** 2026-10-14

**Authors:** Na Ni, Yuting Wang, Qi Wang, Jiajia Ding, Tong Liu, Bixia Zhao

**Affiliations:** 1 School of Nursing, Affiliated Nanhua Hospital, Hengyang Medical School, University of South China, Hengyang, Hunan, China; 2 Department of Neurosurgery, First Affiliated Hospital of University of South China, Hengyang, Hunan, China; Touro University California College of Pharmacy, UNITED STATES OF AMERICA

## Abstract

The Identity, Situation, Background, Assessment, Recommendation (ISBAR) framework is used to standardize clinical handovers. Effective ISBAR implementation requires both training and objective assessment of handover. In Chinese clinical settings, handover quality is commonly evaluated through self-reports or locally developed checklists, which provide limited information about the completeness and accuracy of information transfer. The absence of validated Chinese observational instruments restricts the identification of communication deficiencies, benchmarking of handover performance, and development of targeted workforce training and patient safety interventions. This study aimed to conduct a cross-cultural adaptation and psychometric validation of the ISBAR Structured Handover Observation Tool and evaluate its applicability in Chinese clinical practice. The study was conducted in two phases: cross-cultural adaptation and psychometric evaluation in clinical settings. Content validity was assessed using the content validity index (CVI). Inter-rater reliability was evaluated using the intraclass correlation coefficient (ICC) in a two-way mixed-effects model with absolute agreement. Known-groups validity was examined using the Mann–Whitney U test to compare scores across nurses with varying levels of experience. A total of 233 ICU-to-general ward handovers involving 84 nurses were observed and evaluated. The scale demonstrated good content validity, with item-level CVIs ranging from 0.86 to 1.00 and a scale-level CVI/Ave of 0.98. Based on 50 randomly selected handovers, inter-rater reliability was high, with an ICC of 0.885 for single-rater assessments and 0.939 for average-rater assessments. Known-groups validity analysis showed that nurses with more experience had significantly higher total scores than those with less experience (Z = −4.772, p < 0.001). The Chinese version tool demonstrates satisfactory validity and reliability for evaluating structured handover performance. The tool may be used to identify communication gaps, support competency-based training, monitor handover performance, and provide evidence for patient safety initiatives, facilitating its integration into nursing management and patient safety programs in Chinese clinical settings.

## Introduction

Effective clinical handover is critical to patient safety, ensuring continuity of care and the accurate transmission of clinical information [1–3]. Communication errors during handovers have been identified as a major cause of adverse events in healthcare settings [4]. This is especially true during high-risk transfers, such as when moving patients from the intensive care unit (ICU) to a general ward [5]. The Joint Commission reports that communication breakdowns are the primary root cause of sentinel events, accounting for a significant proportion of serious medical errors [6]. These referral processes involve complex clinical situations, a high volume of information, and changes in nursing responsibilities, which increase the risk of information loss and communication errors [7].

To improve the quality and consistency of clinical communication, structured communication tools such as the Identity, Situation, Background, Assessment, Recommendation ISBAR framework have been widely recommended [6,8]. ISBAR provides a structured framework for organizing and conveying critical patient information [9–12], and previous studies have confirmed its effectiveness in improving communication quality and reducing adverse events. However, the effectiveness of structured communication tools depends on whether they are applied consistently and accurately in clinical practice. Evidence suggests that without systematic evaluation and feedback, adherence to structured communication protocols may decline over time [13]. Therefore, objective and reliable methods are needed to assess whether ISBAR is being applied correctly during clinical handovers.

To address this need, Tangvik et al. [1] developed the ISBAR Structured Handover Observation Tool, which was specifically designed to assess the use of ISBAR during patient transfers from intensive care units to general medical wards. Unlike general communication assessment tools, this tool focuses on whether predefined key information elements are reported, thereby enabling an objective evaluation of adherence to structured communication protocols [1,7,14]. However, evaluating only the completeness of information may not fully reflect the quality of the handover; a complete handover is not necessarily an effective one, as the latter also involves dimensions such as clarity of explanation and interactive confirmation. Previous handover assessment approaches have reflected a methodological trade-off between objectivity and comprehensiveness. Content-based observational tools provide standardized criteria and stronger inter-rater reliability but may not fully capture broader aspects of communication quality, whereas global rating approaches may provide more comprehensive assessments but are more susceptible to assessor subjectivity [2,15]. Given that our aim was to objectively evaluate adherence to structured information transfer, we selected the ISBAR observation tool for cross-cultural adaptation and psychometric validation.

In addition, the implementation and evaluation of structured handover tools are influenced by the cultural and organizational context in which clinical communication occurs. In Chinese healthcare settings, hierarchical organizational structures, high clinical workload, and established communication practices may influence how structured communication frameworks are implemented during clinical handovers. Traditional hierarchical structures may influence nurses’ willingness to actively express concerns, clarify uncertainties, or provide recommendations during interprofessional communication [16]. Meanwhile, the complexity of ICU-to-ward transfers and heavy clinical workloads may increase reliance on experience-based communication rather than standardized information exchange [17]. Therefore, observational tools developed in Western healthcare systems may not fully capture handover behaviors in Chinese clinical environments without appropriate cultural adaptation and psychometric evaluation.

Existing assessment tools have been primarily developed and validated in non-Chinese contexts, with no validated Chinese versions currently available [18,19]. This gap limits the ability to objectively assess the ICU-to-ward handover processes in clinical settings in China. Furthermore, the applicability of these tools in routine clinical practice has not been fully explored. Given the complexity and diversity of ICU-to-ward transfers, a culturally adapted and methodologically sound observational tool is needed to support standardized assessment and quality improvement. Beyond its role as a measurement instrument, a validated Chinese tool could serve as a practical resource for clinical governance by identifying communication vulnerabilities, informing targeted training interventions, and supporting continuous monitoring of handover quality. Therefore, this study aimed to adapt the ISBAR Structured Handover Observation Tool and evaluate the reliability and validity evidence of its Chinese version, thereby providing a validated instrument for objectively assessing structured information transfer and supporting evidence-based quality improvement and patient safety initiatives in Chinese clinical settings.

## Method

### Study design

This methodological study employing a cross-sectional design was conducted in two phases: the cross-cultural adaptation and psychometric validation of the ISBAR Structured Handover Observation Tool.

### Study setting

This study was conducted in the ICUs and general medical wards of two tertiary Grade A hospitals in Hengyang, Hunan Province, China. Data were collected during the routine clinical handover process when patients were transferred from the ICU to the general medical ward, providing an accurate reflection of clinical practice.

### Participants and sampling

The study subjects were nurses involved in the handover process from the ICU to general medical wards.

A total of 246 ICU handover events were collected during the study period. Among these, 52 handover events were audio-recorded for the purpose of inter-rater reliability assessment, while the remaining events were assessed through direct observation only. Thirteen handover events were excluded from the overall dataset because they did not meet the study eligibility criteria or could not be adequately assessed. Specifically, exclusions included transfers from general wards or the emergency department to the ICU (n = 6), transfers from the ICU to surgical wards (n = 4), and handovers that were too brief to permit complete assessment (n = 3). Consequently, 233 eligible ICU-to-general medical ward handover events involving 84 nurses were included in the final analysis. No missing data were identified among the included nurses or handover events.

For inter-rater reliability analysis, the 52 audio-recorded handovers were independently reviewed and scored by two raters. Prior to analysis, two recordings were excluded: one because the recording was interrupted and did not allow adequate assessment of verbal communication behaviors, and the other because the audio file contained no usable sound. Ultimately, 50 recordings were included in the ICC analysis.

The inclusion criteria for the study were as follows: (1) Nurses must hold a valid nursing license and must have worked in the department for at least six months; (2) The patient was transferred from the ICU to a general medical ward; (3) The nurses should have participated in routine handover tasks during the study period and voluntarily agreed to participate in this study; (4) The handover process was completed, allowing for full observation and documentation. The exclusion criteria were as follows: (1) Intern nurses, visiting nurses, or those who had worked in the department for less than six months; (2) The handover process was incomplete or interrupted; (3) The patient was transferred out of the department to a non-ICU unit or transferred into a department other than a general medical ward.

To evaluate known-groups validity, the nurses were divided into two groups based on their years of service: the junior group (years of service < 5 years, n = 44) and the senior group (years of service ≥ 5 years, n = 40). In Chinese nursing research literature, junior nurses are commonly defined as those with five years or less of work experience [20]; previous studies have confirmed that the number of years of experience is significantly associated with communication quality [21]. The Mann-Whitney U test [22] was used to compare ISBAR scores between the two groups, with the expectation that the senior group’s scores would be significantly higher. The baseline characteristics of the two groups, including age, professional title, and educational background, are presented in detail in the results section.

### Instrument

The ISBAR Structured Handover Observation Tool, originally developed by Tangvik et al. [1], is an observation checklist designed to assess the completeness of information transfer during patient handovers.

Based on the ISBAR framework, the tool comprises five dimensions: identification, situation, background, assessment, and recommendation. The 26 items are distributed across these dimensions, with each item representing a key piece of information that should be communicated during the handover process.

Each item is scored using a binary scale (0 = absent, 1 = present) based on whether the relevant information was reported. The total score is calculated by summing the scores of all items; a higher score indicates greater completeness of information transfer.

We obtained permission from the original authors to publish our adapted Chinese version under the CC BY 4.0 license. The scale is available as S1 File.

### Cross-cultural adaptation

The cross-cultural adaptation of the ISBAR Structured Handover Observation Tool was conducted in accordance with the established guidelines proposed by Beaton et al. [23]. Formal permission from the original authors was obtained prior to the adaptation process. The authors also granted permission to translate, adapt, and publish the Chinese version of the tool as supplementary material. The signed permission form is provided as S2 File.

The adaptation procedure consisted of five steps to ensure semantic, conceptual, and cultural equivalence between the original and Chinese versions:

1. Forward translation: Two bilingual nursing researchers independently translated the original instrument into Chinese. This process yielded two initial Chinese versions (T1 and T2).
2. Consolidation: The two translations were compared and consolidated into a preliminary Chinese version (V1). Discussions were held to reach a consensus and resolve discrepancies.
3. Back-translation: Two translators, unaware of the original scale’s content, independently back-translated the consolidated version (V1) into English. This generated two back-translated versions (RT1 and RT2), which were subsequently integrated into a single version.
4. Expert Panel Review: A seven-member expert panel was convened to assess the conceptual and cultural equivalence of the translated version. The panel consisted of four critical care nursing experts (two Associate Chief Nurses and two Chief Nurses), two linguistics experts specializing in medical translation, and one nursing researcher with expertise in nursing methodology and instrument evaluation. The experts had diverse expertise in critical care nursing, nursing quality management, structured communication, medical translation, and instrument evaluation, with all members having over 20 years of experience in their respective fields. Detailed characteristics of the expert panel are presented in Supplementary Table S4. The panel assessed the conceptual equivalence of the back-translated version with the original scale and, through discussion and consensus, produced the pre-final Chinese version (V2).
5. Pilot Testing: In a clinical setting, the pre-final version was pilot-tested during 15 nurse-led patient handover processes to assess its clarity, feasibility, and comprehensibility. Each handover was independently evaluated by two trained assessors. Based on their feedback and observations, the text was fine-tuned to produce the final Chinese version.

Detailed records of the translation and adaptation process are available in Supplementary S3 Dataset.

### Data collection

Data were collected from November 26, 2025 to March 15, 2026. In routine clinical settings, handover processes during patient transfers from the ICU to general medical wards were observed using the ISBAR Structured Handover Observation Tool. To ensure objectivity, the evaluators did not participate in patient care or the handover process.

Both evaluators were experienced clinical nurses with over 10 years of experience in either the ICU or general medical wards. Prior to data collection, the two evaluators received a standardized two-hour training program conducted by the research team. The training consisted of four components: (1) a 20-minute introduction to the theoretical background and five components of the ISBAR framework; (2) a 40-minute item-by-item explanation of the scale, including operational definitions, scoring rules, and examples of acceptable and unacceptable handover behaviors; (3) a 30-minute guided practice session using three standardized handover recordings with discussion of discrepancies; and (4) a 30-minute independent scoring exercise using additional sample recordings followed by feedback and clarification. A scoring manual containing definitions of each item and scoring examples was provided to both evaluators. After completing the training, the evaluators independently scored three pilot handover recordings that were not included in the final dataset. Inter-rater reliability was assessed using the ICC, and the agreement reached a good level (ICC > 0.80), indicating adequate evaluator competence before formal data collection. Any disagreements identified during the pilot scoring were discussed to further clarify the interpretation of scoring criteria.

For the 50 handover recordings selected for inter-rater reliability analysis, both raters independently reviewed and scored the recordings without consultation. The ICC was calculated using the original independent ratings. After independent scoring was completed, any discrepancies in item interpretation were discussed to clarify the application of the scoring criteria and maintain consistency throughout the study. These discussions did not alter the original ratings used for reliability analysis.

For pilot testing (15 handover events) and the 52 handover events selected for inter-rater reliability assessment, handover processes were video-recorded and scored afterward to ensure that observation did not interfere with clinical practice. The remaining handover events were assessed through direct observation by the primary evaluator.

### Data analysis

All statistical analyses were performed using SPSS Version 26.0 (IBM, Armonk, NY, USA).

The ISBAR Structured Handover Observation Tool is a formative measurement instrument rather than a reflective scale. According to COSMIN guidelines, structural validity and internal consistency are not required for formative models, as the items represent distinct components of handover communication rather than manifestations of a single latent construct. Therefore, our psychometric evaluation focused on content validity, inter-rater reliability, and known-groups validity.

Content validity was assessed using the content validity index (CVI). Inter-rater reliability was assessed using the ICC, based on a two-way mixed-effects model of absolute agreement [24]. Known-groups validity was evaluated by comparing ISBAR scores between junior nurses (<5 years of experience) and senior nurses (≥5 years of experience). Because the total score was derived from dichotomous item ratings and did not satisfy the assumptions required for parametric testing, the Mann–Whitney U test was used for group comparisons [22]. Effect sizes (r) were calculated as r = Z/√N, where Z represents the standardized test statistic and N represents the total number of participants.

A p-value < 0.05 was considered statistically significant.

For the inter-rater reliability analysis, 50 handover events were randomly selected from the full dataset of 233 eligible events. The sample size was considered adequate based on methodological recommendations indicating that approximately 30–50 observations are generally sufficient to obtain stable estimates of inter-rater reliability and ICC values [25,26]. For known-groups validity, the sample size of 84 nurses (44 junior and 40 senior) is sufficient to detect a medium-to-large effect (Cohen‘s d ≥ 0.5) with 80% power at α = 0.05. The raw data are available in Supplementary Data S5 Dataset.

### Ethical considerations

This study was approved by the Medical Ethics Committee of the University of South China (Approval No.: 2025−0148) and was conducted in accordance with the Declaration of Helsinki. The approved protocol included video recording of selected handover events for pilot testing and inter-rater reliability assessment.

Written informed consent was obtained from all participating nurses. For video-recorded handovers, patients were also informed and provided written consent; no waiver of consent was recorded. To protect confidentiality, the camera was positioned to avoid capturing patients’ faces or identifying information. Recordings were stored on a USB drive accessible only to the research team, used exclusively for scoring, and permanently deleted after scoring. All data were anonymized prior to analysis.

## Results

### Cross-cultural adaptation

The translation and back-translation processes were completed smoothly without encountering any significant difficulties, and the overall structure of the original scale was preserved.

Based on the expert panel’s assessment, minor adjustments were made to the scale to enhance semantic clarity and cultural relevance. These revisions primarily involved optimizing the wording and modifying the item descriptions to better reflect local clinical practice. Detailed records of the translation, adaptation, and expert consultation can be found in Supplementary Data S3 Dataset.

Seven experts participated in two rounds of expert consultation. Following expert consultation and consensus discussions, one item related to communication regarding community services was deleted. Most experts noted that, in the Chinese clinical context, community-based healthcare services have not yet been widely implemented, which limits the applicability of this item in routine handovers. Furthermore, factors such as nursing needs and social support were considered to be adequately covered by other items, potentially leading to content duplication. Therefore, the expert panel ultimately agreed to delete this item to enhance the scale’s cultural relevance and overall feasibility.

Following expert consultation and cultural adaptation, the final Chinese version of the ISBAR Structured Handover Observation Tool retained the original five-dimensional structure and consisted of 25 items.

### Content validity

In the first round of expert consultation, the item-level content validity indices (I-CVI) ranged from 0.57 to 1.00, with S-CVI/UA at 0.62 and S-CVI/Ave at 0.93.

Following revisions, the results from the second round improved, with I-CVI ranging from 0.86 to 1.00, S-CVI/UA of 0.84, and S-CVI/Ave of 0.98. Detailed records of the expert consultation are available in Supplementary Data S6 Dataset.

These results indicate that the content validity of the scale is at an acceptable to excellent level.

### Reliability

#### Characteristics of case handover.

A total of 50 eligible transfer cases were included in the inter-rater reliability analysis. The mean age of the patients was 68.6 years (standard deviation = 10.43), and the mean length of stay in the ICU was 3.14 days (standard deviation = 1.58). The mean total scores assigned by the two raters were comparable (19.50 ± 3.65 vs. 19.08 ± 3.52), indicating good inter-rater agreement. Patients were transferred to multiple departments, with the majority admitted to the Department of Gastroenterology (32.0%), followed by the Department of Neurology (20.0%), the Department of Cardiology (18.0%), and the Department of Pulmonology (16.0%).

#### Inter-rater reliability.

The ISBAR observation tool demonstrated good inter-rater reliability. The single-measurement ICC for the total score was 0.885 (95% CI: 0.795–0.935), and the average-measurement ICC was 0.939 (95% CI: 0.886–0.966). The single-measurement ICC values for each dimension ranged from 0.789 to 0.941, with the reliability for all dimensions ranging from good to excellent (Table 1).

**Table 1:** Reliability of the ISBAR Structured Handover Observation Tool.

Dimension	ICC (single)	95% CI
Total	0.885	0.795–0.935
Identification	0.941	0.898–0.966
Situation	0.913	0.853–0.950
Background	0.917	0.858–0.952
Assessment	0.789	0.634–0.880
Recommendation	0.799	0.671–0.880

*ICC, intraclass correlation coefficient; CI, confidence interval. ICC values ≥ 0.75 indicate good reliability, and ≥ 0.90 indicate excellent reliability.*

### Validity

#### Baseline characteristics of nurses.

This study included a total of 84 nurses, with 44 in the junior group (i.e., less than five years of experience) and 40 in the senior group (i.e., five or more years of experience). The distribution of age, gender, professional title, educational background, and the number of handover sessions attended in the past month for both groups is shown in Table 2.

**Table 2:** Baseline characteristics of nurses by experience group.

Characteristic	Low-experience (n = 44)	High-experience (n = 40)	Total (N = 84)	χ² (df)	p-value
Age, n (%)				24.180 (3)	<0.001†
≤25 years	16 (36.4)	0 (0.0)	16 (19.0)		
26-30 years	21 (47.7)	21 (52.5)	42 (50.0)		
31-35 years	7 (15.9)	12 (30.0)	19 (22.6)		
≥36 years	0 (0.0)	7 (17.5)	7 (8.3)		
Gender, n (%)				8.505 (1)	0.004‡
Male	18 (40.9)	5 (12.5)	23 (27.4)		
Female	26 (59.1)	35 (87.5)	61 (72.6)		
Professional title, n (%)				21.422 (2)	<0.001
Nurse	19 (43.2)	1 (2.5)	20 (23.8)		
Senior nurse	20 (45.5)	24 (60.0)	44 (52.4)		
Supervisor nurse	5 (11.4)	15 (37.5)	20 (23.8)		
Education, n (%)				2.161 (2)	0.339§
Associate degree	6 (13.6)	2 (5.0)	8 (9.5)		
Bachelor’s degree	37 (84.1)	36 (90.0)	73 (86.9)		
Master’s degree	1 (2.3)	2 (5.0)	3 (3.6)		
Handovers per month, n (%)				0.033 (2)	0.984
≤5	13 (29.5)	12 (30.0)	25 (29.8)		
6-10	26 (59.1)	23 (57.5)	49 (58.3)		
11-20	5 (11.4)	5 (12.5)	10 (11.9)		

†Two cells (25.0%) have expected count <5 (minimum 3.33).^‡^ Fisher’s exact test p = 0.006 (2-tailed).^§^ Four cells (66.7%) have expected count <5 (minimum 1.43); result should be interpreted with caution

The distribution of handover events across nurses and hospitals was examined. A total of 233 handover events were collected from 84 nurses, with a mean of 2.77 observations per nurse (median = 2, range = 1–11). Hospital A contributed 120 handover events involving 43 nurses, whereas Hospital B contributed 113 handover events involving 41 nurses.

### Comparison between hospitals

To explore potential site effects, ISBAR total scores were compared between the two participating hospitals using the Mann–Whitney U test. No statistically significant difference was observed between hospitals (U = 775.500, Z = −0.949, p = 0.343), indicating comparable handover performance across study sites.

### Known-groups validity

Known-groups validity was assessed by comparing handover scores among nurses with different levels of clinical experience.

The results showed that nurses with more clinical experience had significantly higher total scores than those with less experience (Z = −4.772, p < 0.001, r = 0.521), indicating a large effect size.

At the dimensional level, statistically significant differences were observed in the “Identification” (Z = −2.241, p = 0.025), “Background” (Z = −3.593, p < 0.001), and “Assessment” (Z = −3.628, p < 0.001) dimensions. In contrast, no statistically significant differences were found in the “Situation” (Z = −1.451, p = 0.147) and “Recommendation” (Z = −1.785, p = 0.074) dimensions (Table 3).

**Table 3:** Known-groups validity.

	Total score	I	S	B	A	R
Mann–Whitney U	347.500	641.500	719.500	482.500	476.000	688.500
Wilcoxon W	1337.500	1631.500	1709.500	1472.500	1466.000	1678.500
Z	−4.772	−2.241	−1.451	−3.593	−3.628	−1.785
Asymp. Sig. (2-tailed)	<0.001	0.025	0.147	<0.001	<0.001	0.074
Effect size (r)	0.521	0.244	0.158	0.392	0.396	0.195

These findings indicate that the scale is capable of distinguishing between groups that would theoretically be expected to differ in handover performance, thereby providing evidence for known-groups validity.

## Discussion

This study conducted a cross-cultural adaptation and psychometric validation of the ISBAR Structured Handover Observation Tool. The results indicate that the Chinese version of the scale demonstrates good content validity (S-CVI/Ave = 0.98) and inter-rater reliability (total score ICC = 0.885, 95% CI: 0.795–0.935) and is effective in distinguishing nurses with different levels of clinical experience (Z = −4.772, p < 0.001). These findings support the tool’s applicability in assessing the completeness of information transfer during patient handovers from the ICU to general medical wards.

In terms of content validity, the S-CVI/Ave for this scale was 0.98, which is higher than the S-CVI value (0.91) reported by Tangvik et al. [1] in the original study. This indicates that the Chinese version maintains good content validity following cross-cultural adaptation and demonstrates high inter-rater reliability. Regarding inter-rater reliability, the original study did not report this metric; however, this study found that the ICC for the total score was 0.885 (95% CI: 0.795–0.935), with ICCs for each dimension ranging from 0.789 to 0.941, providing new evidence of the instrument’s reliability. Furthermore, the known-groups validity results of this study showed that nurses with higher seniority scored significantly higher than those with lower seniority (Z = −4.772, p < 0.001), which is consistent with previous research: He et al. [21] found that years of service were a significant predictor of nurses’ clinical communication skills (F = 56.212, p < 0.001), while Qiu et al. [27] also reported a positive correlation between years of experience and medical narrative competence. These findings suggest that the ISBAR observation tool is capable of distinguishing between groups that would theoretically be expected to differ in handover performance, thereby providing evidence for known-groups validity.

However, not all dimensions showed statistically significant differences. The “Situation” and “Recommendation” dimensions did not differ between experience groups. This may be due to the standardized, procedural nature of Situation reporting [28], the limited number of Recommendation items, and the dichotomous scoring that captures presence rather than quality of information [29]. In the Chinese hospital context, the hierarchical communication culture may further attenuate experience-related differences in these dimensions—factual Situation information is often documented and reported procedurally, leaving limited room for senior nurses to demonstrate clinical reasoning, while Recommendation expression requires nurses to actively communicate clinical judgments and anticipate potential risks. However, within hierarchical communication environments, junior nurses may be less likely to initiate recommendations or challenge existing plans, as decision-making authority is often perceived to reside with senior clinicians or physicians. Consequently, differences in clinical reasoning ability between nurses with different levels of experience may not be fully reflected through observable handover behaviors. These factors may mask the potential expertise gap in these specific dimensions. As the two groups also differed in age, gender, and professional title, these findings should be interpreted as evidence of known-groups validity rather than a direct effect of clinical experience.

The pattern of item-level performance showed both similarities and differences compared with previous international ISBAR studies. Lower Identification compliance has also been reported in other countries [30], suggesting that incomplete identification reporting may be a common challenge in structured handovers. In the Chinese ICU-to-ward context, this may also be influenced by local workflow, as patient identity is often confirmed before transfer through telephone handover or electronic systems and further verified by receiving nurses. In contrast, the lower Recommendation compliance observed in our study differed from the high compliance reported in Iran (91%–96%) [30], which may reflect differences in nursing communication norms, professional autonomy, and hierarchical structures across healthcare systems.

Beyond its psychometric properties, the tool may have practical value for clinical governance. Because the instrument provides item- and dimension-level information on handover performance, it enables nurse managers to identify recurring communication deficiencies during ICU-to-ward transfers and to develop targeted educational interventions. Dimensions with consistently lower scores may indicate areas requiring additional training, supervision, or process improvement. The tool may also support routine auditing of handover practices and facilitate feedback mechanisms aimed at strengthening adherence to structured communication standards. In the future, integration of the tool into electronic quality management systems may further facilitate continuous monitoring and feedback of handover performance.

This study has several limitations. First, although dichotomous scoring effectively assesses whether information was conveyed, it does not reflect the quality or depth of that transmission. Previous studies have noted that while dichotomous checklists can distinguish between operators of different skill levels, they cannot provide detailed feedback [29]. Second, the “Recommendations” dimension includes only two items, which may limit its statistical discriminatory power. Third, the sample was drawn from two tertiary hospitals in one region of China, and although no significant between-hospital differences were found, generalizability to other settings requires further validation. Fourth, some nurses contributed to multiple handover events, introducing potential within-nurse correlation; future studies may consider multilevel modeling to address this. Finally, the Hawthorne effect may have transiently elevated scores due to awareness of observation [31].

In conclusion, the Chinese version of the ISBAR Structured Handover Observation Tool demonstrates good reliability and validity in assessing patient handover from the ICU to general medical wards and may support structured communication assessment and quality improvement in Chinese clinical settings.

## Conclusions

This study successfully adapted the ISBAR Structured Handover Observation Tool for the Chinese clinical context, and provided evidence regarding the psychometric properties of its Chinese version for assessing ICU-to-ward patient handovers. The Chinese version of the scale demonstrated good content validity, inter-rater reliability, and known-groups validity, supporting its use as an objective tool for evaluating the completeness of information transfer based on the ISBAR framework. In clinical practice, the tool offers a practical means of moving beyond retrospective documentation toward prospective quality monitoring and targeted training interventions. Future efforts should explore the integration of this observational tool into digital healthcare platforms or electronic health record systems as a quality monitoring module, enabling real-time assessment, automated feedback, and continuous improvement of handover practices. Such digital integration may facilitate early identification of communication gaps, support targeted training interventions, and ultimately contribute to reducing communication-related sentinel events during high-risk ICU-to-ward transfers.

## Supporting information

S1 File Observation checklist. Chinese version of the ISBAR structured handover observation tool. (PDF)

S2 File Permission form. Signed permission from the original authors. (PDF)

S3 Dataset Translation and cross-cultural adaptation records. (XLSX)

S4 Table Demographic and Professional Characteristics of the Expert Panel. (XLSX)

S5 Dataset Raw data of ISBAR observation scores and nurse characteristics. (XLSX)

S6 Dataset Expert consultation records for content validity evaluation. (XLSX)
