# Supplementary material for: Cross-cultural adaptation and psychometric validation of the ISBAR structured handover observation tool for ICU-to-ward patient transfer in China
Source: PLoS One. 2026 Oct 14;21(10):e0347947. doi: 10.1371/journal.pone.0347947 (PMC13667774; doi:10.1371/journal.pone.0347947)
Supplement: Supplementary file 1 [file pone.0347947.s001.pdf]

维度	条目	是（有做到/有提及）	否（没有做到/没有提及）	在询问下才说明（即接受方追问后才补充）	不适用/无相关（比如患者确实没有改情况，不需要交接）	备注/评论
身份识别	1. 说明患者姓名	☐	☐	☐	☐	
	2. 说明患者年龄	☐	☐	☐	☐	
状况	3. 说明入院时间	☐	☐	☐	☐	
	4. 说明入院主诉	☐	☐	☐	☐	
	5. 简要说明已实施的主要治疗和护理方案	☐	☐	☐	☐	
	6. 说明传染情况及隔离类型	☐	☐	☐	☐	
	7. 说明当前病情诊断	☐	☐	☐	☐	
背景	8. 说明主要既往病史	☐	☐	☐	☐	
	9. 交接关键用药清单（如药物名称、剂量、给药途径及频次）	☐	☐	☐	☐	
	10. 说明过敏史	☐	☐	☐	☐	
	11. 说明异常心理及认知状态	☐	☐	☐	☐	
	12. 说明躯体功能水平及活动能力	☐	☐	☐	☐	
	13. 说明家属特殊状况（如未成年子女照护、关键决策者、老年或认知障碍家属等）	☐	☐	☐	☐	
评估	14. 说明呼吸系统观察指标（血氧饱和度、呼吸频率、痰液性状、血气分析）	☐	☐	☐	☐	
	15. 说明呼吸系统支持措施（氧疗方式、吸入治疗、正压呼气/呼气末正压通气、肺部物理治疗）	☐	☐	☐	☐	

	16. 说明循环系统观察指标（血压、心率、体温、尿量）	☐	☐	☐	☐	
	17. 说明循环系统支持措施（静脉通路、静脉治疗及药物输入情况）	☐	☐	☐	☐	
	18. 说明意识水平及神经系统观察指标（格拉斯哥昏迷指数(GCS)/AVPU 量表、瞳孔反应、MR/CT）	☐	☐	☐	☐	
	19. 说明排泄状况及相关治疗	☐	☐	☐	☐	
	20. 说明营养状况（饮食、营养评分及治疗）	☐	☐	☐	☐	
	21. 说明疼痛情况（疼痛评分及镇痛治疗）	☐	☐	☐	☐	
	22. 说明伤口情况或压力性损伤风险	☐	☐	☐	☐	
	23. 补充未提及的检查或检验	☐	☐	☐	☐	
建议	24. 提出后续的护理建议	☐	☐	☐	☐	
	25. 说明治疗限制措施（如：拒绝心肺复苏(DNR)、限制有创通气等）	☐	☐	☐	☐	
