# Supplementary material for: Cross-cultural adaptation and psychometric validation of the ISBAR structured handover observation tool for ICU-to-ward patient transfer in China
Source: PLoS One. 2026 Oct 14;21(10):e0347947. doi: 10.1371/journal.pone.0347947 (PMC13667774; doi:10.1371/journal.pone.0347947)
Supplement: Supplementary file 2 [file pone.0347947.s002.pdf]

Gmail **NINA <n37744141@gmail.com>**

Request for Permission to Translate, Culturally Adapt, and Use the ISBAR Communication Scoring Tool

3 封邮件

NINA <n37744141@gmail.com> 2025年6月14日 21:06
收件人："lindaskaugtangvik@gmail.com" <lindaskaugtangvik@gmail.com>

Dear Dr. Linda Skaug Tangvik,
I hope this message finds you well. My name is NI NA, and I am a postgraduate student at the University of South China, located in Hunan Province, China. My research focuses on critical care and emergency nursing, with a current emphasis on optimizing the in-hospital transfer process for trauma patients.
I recently came across your "Observasjonsskjema for måling av ISBAR" published in *Inspira*, as well as your article titled *"Intrahospital handovers before and after the implementation of ISBAR communication: A quality improvement study on ICU nurses' handovers to general medical ward nurses"* in *Nursing Reports*. I found your work highly insightful and potentially very valuable in the context of Chinese nursing practice.
I am writing to kindly request your permission to translate and culturally adapt the ISBAR communication scoring tool into Chinese, for use with Chinese-speaking participants. I plan to follow a rigorous cross-cultural adaptation process, including

1. Forward translation by bilingual experts
2. Backward translation to ensure conceptual equivalence
3. Cognitive debriefing with target users
4. Validation of the translated version through a pilot study

Additionally, I would like to inquire whether a Chinese translation of the ISBAR communication scoring tool already exists or is currently under development by any other researchers or institutions. If so, I would greatly appreciate it if you could share any relevant information or suggest a possible collaboration.
If you agree, I would also like to ask whether there are any formal procedures or licensing requirements I should follow to obtain your permission and ensure appropriate usage.
Thank you very much for your time and consideration. I would be honored to receive your guidance and support, and I look forward to your response.
Warm regards,
NI NA
Postgraduate Student
School of Nursing, University of South China
Hunan Province, China
n37744141@gmail.com

Linda Skaug Tangvik <lindaskaugtangvik@gmail.com> 2025年6月18日 05:28
收件人：NINA <n37744141@gmail.com>

Dear NINA,

Thank you for your email. I am honored that you are interested in my work on ISBAR. I have talked with my co-authors about your request, and we give you permission to translate and culturally adapt the ISBAR communication scoring tool into Chinese. I am not aware of any formal procedures we need to follow to grant you permission, all we expect is that you use a reference to our article when you mention the original tool. We would also appreciate an update when you publish your study, so that we can read it.

Good luck on your research. It is exciting that the ISBAR communication tool keeps spreading worldwide. Don`t hesitate to contact med again if you need more information.

Kind regards
Linda Skaug Tangvik
MNSc, Intensive care nurse

[引用文字已隐藏]

NINA <n37744141@gmail.com> 2025年6月19日 10:27
收件人：Linda Skaug Tangvik <lindaskaugtangvik@gmail.com>

Dear Dr. Tangvik,

I am truly delighted to receive your reply. Thank you very much for granting me the permission to translate and culturally adapt the ISBAR communication scoring tool into Chinese.

I will strictly follow your request by citing your original article when referring to the tool, and I will make sure to share the published results of my study with you once it is completed. I sincerely appreciate your generosity and support.

Thank you again for your kind permission. It means a lot to me and my research.

Best regards
NINA

Linda Skaug Tangvik <lindaskaugtangvik@gmail.com> 于2025年6月18日周三 05:27写道：
[引用文字已隐藏]
